# The effect of opportunistic illness on HIV RNA viral load and CD4+ T cell count among HIV-positive adults taking antiretroviral therapy

**DOI:** 10.7448/IAS.16.1.17355

**Published:** 2013-04-01

**Authors:** John P Ekwaru, James Campbell, Samuel Malamba, David M Moore, Willy Were, Jonathan Mermin

**Affiliations:** 1 Division of Epidemiology, School of Public Health, University of California, Berkeley, CA, USA; 2 CDC-Uganda and Uganda Virus Research Institute, Entebbe, Uganda; 3 British Columbia Centre for Excellence in HIV/AIDS, Vancouver, BC, Canada; 4 Faculty of Medicine, University of British Columbia, Vancouver, BC, Canada; 5 Division of HIV/AIDS Prevention, National Center for HIV, Viral Hepatitis, Sexually Transmitted Diseases and Tuberculosis Prevention, Centers for Disease Control and Prevention, Atlanta, GA, USA

**Keywords:** ART, CD4+ T cell lymphocyte count, HIV RNA viral load, HIV, opportunistic infections, Uganda

## Abstract

**Introduction:**

HIV RNA viral load (VL) has been shown to increase during opportunistic illnesses (OIs), suggesting active HIV replication in response to infection among patients not taking antiretroviral therapy (ART). We assessed the effects of OIs on HIV RNA VL and CD4+ T cell counts among patients on ART with initially suppressed VL.

**Methods:**

Between 2003 and 2007, we enrolled and followed 1094 HIV-1-infected adults who initiated ART and had quarterly blood draws for VL and CD4+ T cell count. In VL/CD4+ T cell measurement intervals following undetectable VL, we compared the elevation in VL to detectable levels and CD4+ T cell count changes between intervals when participants had episodes of OIs and intervals when they did not have OIs.

**Results:**

VL was more likely to be detectable if participants had OIs in the prior three months compared to when they did not (OR=4.0 (95% CI=1.9–8.6)). The CD4+ T cell counts declined 24.1 cells/µL per three months in intervals where the participants had OIs compared to an increase of 21.3 cells/µL per three months in intervals where they did not have OIs (adjusted difference in the rate of CD4+ T cell count change of 61.7 cells/µL per three months (95% CI=13.7–109.7), P value=0.012). The rate of CD4+ T cell count increase was 25.6 cells/µL per three months (95% CI=11.6–39.6) higher for females compared to males (*p* value=<0.001), 1.4 cells/µL per three months lower per one year increase in age (*p* value=0.046) and 4 cells/µL per three months lower per 10 cells/µL increase in the starting CD4+ T cell count value (*p* value=<0.001).

**Conclusion:**

Episodes of opportunistic infections among patients taking ART with undetectable VL were associated with elevation of HIV RNA VL to detectable levels and decline in CD4+ T cell counts.

**Clinical Trial Number**: NCT00119093.

## Introduction

Clinical observations in HIV-positive patients show increased plasma HIV viral load (VL) during opportunistic illnesses (OIs), suggesting active HIV replication in response to OIs. Malaria [1,2], tuberculosis, pneumonia and herpes simplex virus have been associated with increased HIV VL and a more rapid decrease in CD4+T cell count among HIV-infected people not taking antiretroviral therapy (ART) [3–7]. Occurrence of OIs has also been shown to be predictive of increased risk of death, independent of CD4+T cell count [8,9]. However, it is not known if OIs have an effect on immune function or viral replication among people taking ART. Theoretical models have suggested that episodes of transient viremia in otherwise well-suppressed chronically infected HIV patients under drug therapy may be triggered by intercurrent infections that cause a rise in activated T cells, and thus transient bursts of CD4+T cell infection and resultant viremia [10]. We evaluated the effect of OIs on HIV RNA VL and CD4+T cell count among HIV-infected people who were taking ART and had initially attained undetectable VL.

## Methods

We analyzed data from the Home-Based AIDS Care Project (HBAC), a three-year randomized trial designed to compare three different monitoring strategies for HIV-positive patients receiving ART. Details of this trial have been presented elsewhere [11].

### Study participants

Between May 2003 and April 2007, we enrolled and followed HIV-1-infected clients of the The AIDS Support Organization (TASO), who were initiated on ART at the Tororo Branch of TASO. Enrolment was offered to clients with a CD4+T cell count<250 cells/µL or severe HIV disease (defined as WHO Stage 3 or 4 or a history of recurrent herpes zoster), an AST or ALT<5 times upper limit of normal, a calculated creatinine clearance≥25 mL/min, and a Karnofsky Score>40%. The Cockroft–Gault formula was used to estimate creatinine clearance. The first-line ART regimen was stavudine, lamivudine and nevirapine (or efavirenz for those taking concurrent rifampicin), and all participants received cotrimoxazole prophylaxis. Pre-packaged ART, cotrimoxazole and other medicines were replaced using a weekly storage container, and pill counts conducted at the study clinic by a pharmacist.

All participants provided written informed consent in their preferred language and were free to withdraw their participation in the study at anytime without losing access to free ART from TASO. The study was approved by Uganda National Council of Science and Technology, the Institutional Review Boards of the Uganda Virus Research Institute, and the Centers for Disease Control and Prevention (CDC). Funding was provided by the U.S. Department of Health and Human Services/CDC through the Emergency Plan for AIDS Relief.

### Data collection methods

Trained lay field officers visited clients’ homes weekly to deliver medications and collect data regarding drug adherence, potential symptoms of drug toxicity or death of a household member in the preceding seven days [11]. Participants were weighed monthly during home visits, and these weights and body mass index scores were systematically provided to clinicians. After enrolment, no routine clinic visits were scheduled, but participants were encouraged to come to the clinic or hospital if they were ill, and were transported to the clinic for assessment if they had specifically defined symptoms or severe illness during a home visit.

CD4+ T cell counts and HIV RNA VL were measured at three-monthly intervals. Field workers completed weekly client monitoring forms that included information on client symptoms, problems with taking medication or other information which might impact participant health. All diagnoses of OIs were presented at the weekly medical case conference and reviewed by the medical team as a whole. For diagnosis of an OI, we considered all WHO Stage 3 or 4 illnesses and we also included malaria. While not technically an opportunistic illness, which usually refers to WHO Stage 3 or 4 illnesses, malaria is one of the most common causes of illness and death among HIV-positive individuals in endemic areas of sub-Saharan Africa. As well malaria infections have been shown to affect HIV VL in HIV-positive individuals not on ART [2].

### Diagnosis and measurement methods

Cryptococcal disease was diagnosed by compatible symptoms and serum cryptococcal antigen testing (Crypto-LA, Wampole Laboratories, Cranberry, NJ). Pulmonary tuberculosis (TB) was defined as two sputum smears positive for acid-fast bacilli using microscopy or negative sputum smears with a chest radiograph compatible with TB and a lack of response to a two-week trial of antibiotic therapy. Extra-pulmonary TB was diagnosed by clinical presentation and infrequently by lymph node biopsy and pathological confirmation. Diagnoses of Kaposi's sarcoma and cervical cancer were based on biopsy results. *Pneumocystis jirovecii* pneumonia was diagnosed clinically using chest radiography, clinical presentation and a response to cotrimoxazole treatment. All illnesses were diagnosed by physicians at the study clinic in Tororo and reviewed at the weekly medical case conferences.

We measured HIV VLs using Cobas Amplicor HIV-1 Monitor version 1.5 (Roche, Branchburg, NJ) and enumerated CD4+ T cell count using TriTEST reagents following an in-house dual platform protocol and MultiSET and Attractors software using an FACScan or FACSCalibur flow cytometer (Becton-Dickinson, Franklin Lakes, NJ).

We estimated adherence using pill count data stored in a computerized pharmacy database as: (number of pills delivered minus number of pills returned) divided by number of pills delivered.

### Data analysis methods

Data were entered using Epi Info (CDC, Atlanta, GA) and analyzed using SAS (SAS Institute, Cary, NC).

To assess the effect of OIs on HIV RNA VL and CD4+ T cell count among participants with suppressed HIV RNA VL, we compared the elevation in HIV RNA VL to detectable levels and CD4+T cell count changes between measurement intervals when participants had episodes of OIs and intervals when they did not have OIs. We based our analysis on quarterly measurement intervals that started with undetectable HIV RNA VL (<50 copies/mL). We conducted analysis with two outcome measures, i.e., detectable HIV RNA VL (>50 copies/mL) in measurements that followed a previous quarterly measurement with undetectable HIV RNA VL, and rate of CD4+ T cell count change within measurement intervals. For the analysis of the effect of OIs on detectable HIV RNA VL, we used a logistic regression model using a log-link function. For the analysis of rate of change in CD4+T cell count, we used a linear regression model. In both models, generalized estimating equation method with an exchangeable correlation structure was used to take into account the dependence of observations due to repeated measures for the same individuals. Covariates that were considered in the models were gender, duration on ART at the start of the interval, age, BMI, ART adherence and first-line ART regimen.

For participants who had an OI following undetectable HIV RNA VL, we plotted the subsequent changes in CD4+T cell count over time, starting from the time of the undetectable HIV RNA VL preceding the OI. For comparison, we also plotted a similar graph for those who never had OIs, starting from the time of their first undetectable HIV RNA VL.

## Results

Of the 1094 antiretroviral-naive clients who were started on ART, 47 had no follow up HIV RNA VL or CD4+ T cell count measurements after initiating ART and were excluded from the analysis. Of the remaining 1047 participants, 73% were female, the median age was 38 years, the median CD4+T cell count at enrolment was 131 cells/µL (IQR=72–196) and median HIV RNA VL at enrolment was 207,787 copies/mL (IQR=69,100–492,000) (Table 1).

**Table 1:** Baseline characteristics of HIV-positive adults who were enrolled between May 2003 and April 2007

Characteristic	*N =*1047	%
Gender		
Female	767	73.3
Male	280	26.7
Education level		
None	562	54.7
Primary	218	21.2
Post-primary	248	24.1
Missing	19	
Median age in years (IQR)	38 (32–43)	
Baseline CD4+ T cell count in cells/µL		
<50	194	18.5
50–200	622	59.4
>200	231	22.1
Median (IQR)	131 (70–196)	
Baseline viral load (copies/mL)		
<1000	8	0.8
1000–9999	42	4.0
10,000–99,999	298	28.5
≥100,000	699	66.8
Median (IQR)	207,394 (69,100–492,000)	
Body mass index (kg/m^2^)		
<18.5	299	29.4
18.5–24.9	661	65.1
25–29.9	46	4.5
≥30	10	1.0
Missing	31	
Median (IQR)	19.8 (18.2–21.5)	
First-line ART regimen		
Efavirenz+lamivudine+stavudine	33	3.2
Nevirapine+lamivudine+stavudine	1014	96.8

The median interval between lab measurements was 91 days, and the 1047 people included in the analysis had a total of 11,477 visits in the four years of follow up where laboratory measurements were done. For 79% of these, the HIV RNA VL in the preceding measurement was undetectable. A total of 176 OIs were diagnosed in 137 individuals during follow up. The five most common diagnoses were malaria (23%), cryptococcal meningitis (21%), TB (19%), Kaposi's sarcoma (12%) and candidiasis (8%). The median time from ART initiation to OI diagnosis was six months, and the median CD4+ T cell count at the time of diagnosis was 109.5 cells/µL. Of the 176 OIs that occurred during follow up, 90 (48%) occurred after the participants had initially achieved an undetectable HIV RNA VL. In 77 (86%) of these 90 OI diagnoses, the immediately preceding measured HIV RNA VL was undetectable (<50 copies/mL), in 5 (6%) it was between 50 and 10000 copies/ mL and in 8 (9%) it was>1000 copies/mL, the definition of virologic failure according to the Ugandan Ministry of Health national antiretroviral treatment guidelines [12].

### Effect of OIs on HIV RNA VL

A total of 9036 quarterly measurements followed a previous measurement for which the HIV RNA VL was undetectable. Of these 9036 measurements, OIs occurred in 56 (0.6%) and VL become detectable in 371 (4%).

The HIV RNA VL was more likely to be detectable if participants had OIs since the previous quarterly measurement, 14% (8/56), compared to when they had none, 4% (363/8980). This difference was statistically significant (OR=3.8 (95% CI=1.73–8.39); *p*=0.001), after adjusting for the non-independence of repeated observations within an individual, gender, duration on ART, age, BMI at baseline, first-line ART regimen, ART adherence preceding the interval and previous three month CD4+ T cell count (Table 2). In a similar analysis, a significant effect of OIs on HIV RNA VL was seen even when a conservative definition of virologic failure of >1000 copies/mL was used (OR=10.8 (4.1 to 28.4); *p=*<0.001)).

**Table 2:** Association between opportunistic illness and HIV RNA viral load elevation to detectable levels

	Female		Male		All	
			
Variable	OR (95% CI)	*p*	OR (95% CI)	*p*	OR (95% CI)	*p*
Had OI in the previous three months	1.53 (0.19–12.65)	0.693	4.87 (2.13–11.16)	<0.001	3.82 (1.73–8.39)	0.001
CD4+ T cell count at start of interval (‘00)	1.11 (1.02–1.20)	0.020	1.03 (0.96–1.10)	0.419	1.06 (1.01–1.12)	0.032
Duration on ART at start of interval (in months)	0.98 (0.96–1.01)	0.143	0.98 (0.97–1.00)	0.029	0.98 (0.97–0.99)	0.005
Age (in years)	0.98 (0.96–1.00)	0.126	1.01 (0.99–1.02)	0.484	1.00 (0.98–1.01)	0.559
BMI at baseline	1.05 (0.97–1.15)	0.246	0.96 (0.93–1.01)	0.090	0.98 (0.95–1.02)	0.289
Adherence preceding the interval	0.71 (0.58–0.88)	0.001	0.87 (0.74–1.03)	0.108	0.81 (0.71–0.92)	0.001
First-line ART regimen						
Efavirenz+lamivudine+stavudine	1.01 (0.33–3.10)	0.979	1.13 (0.56–2.28)	0.740	1.07 (0.60–1.92)	0.818
Nevirapin+lamivudine+stavudine	1.00	ref	1.00	ref	1.00	ref
Female					0.70 (0.54–0.91)	0.007

For individuals that had increases in HIV RNA VL to detectable levels, the median VL achieved was higher among those who had had an OI or malaria (25,500 copies/ mL; IQR=1304–82,800 copies/mL) compared to those who had not had an OI (126 copies/ mL; IQR=68–399 copies/mL).

### Effect of OIs on CD4+ T cell counts

Table 3 shows models for the rate of CD4+T cell count change per three months. CD4+ T cell count decreased at a rate of 24.1 cells/µL per three months in intervals when participants had OIs, compared to an increase of 21.3 cells/µL per three months in intervals when they had no episodes of OIs. After controlling for age, baseline BMI, CD4+ T cell count at the start of the interval, duration on ART, first-line ART regimen and ART adherence, the adjusted difference in the rate of CD4+T cell count change was 60.7 cells/µL per three months (95% CI=12.6–108.8; *p*=0.013), comparing intervals when participants had OIs to intervals when they did not have OIs. Other factors that were significantly associated with the rate of CD4+ T cell count change were previous three-month CD4+T cell count, duration on ART and gender. After controlling for OIs, age, CD4+ T cell count at the start of the interval and BMI, the rate of CD4+ T cell count increase was on average 25.7 cells/µL per three months (95% CI=11.6–39.7) higher for females compared to males (*p=*<0.001). The rate of increase was negatively associated with older age (1.5 cells/µL per three months lower per one year increase in age; *p=*0.044) and CD4+T cell count values at the start of the interval (0.4 cells/µL per three months lower per unit increase in CD4+ T cell count at start of the interval, *p=*<0.001).

**Table 3:** Association between opportunistic illness and change in CD4+ T cell count per three months

	Female		Male		All	
			
Variable	Coefficient (95% CI)	*p*	Coefficient (95% CI)	*p*	Coefficient (95% CI)	*p*
Had OI in the previous three months	−59.0 (−125.6 to 7.5)	0.082	−60.6 (−119.6 to −1.6)	0.044	−60.7 (−108.8 to −12.6)	0.013
CD4+ T cell count at start of interval	−0.9 (−1.0 to −0.7)	<0.001	−0.4 (−0.5 to −0.3)	<0.001	−0.4 (−0.5 to −0.3)	<0.001
Duration on ART at start of interval (in months)	3.3 (2.4 to 4.2)	<0.001	3.9 (0.9 to 6.8)	0.010	3.4 (1.2 to 5.6)	0.003
Age (in years)	−2.0 (−3.9 to −0.2)	0.033	−1.6 (−3.6 to 0.4)	0.112	−1.5 (−2.9 to −0.0)	0.044
BMI at baseline	8.6 (0.8 to 16.4)	0.031	1.5 (−0.8 to 3.9)	0.199	1.8 (−0.3 to 4.0)	0.098
Adherence preceding the interval	−5.2 (−19.0 to 8.6)	0.463	9.9 (−0.3 to 20.1)	0.056	7.0 (−1.1 to 15.1)	0.088
First-line ART regimen						
Efavirenz+lamivudine+stavudine	−9.8 (−71.3 to 51.8)	0.756	−23.0 (−51.7 to 5.6)	0.115	−18.5 (−40.2 to 3.1)	0.093
Nevirapin+lamivudine+stavudine		ref		ref		ref
Female					25.7 (11.6 to 39.7)	<0.001

**Figure 1:**
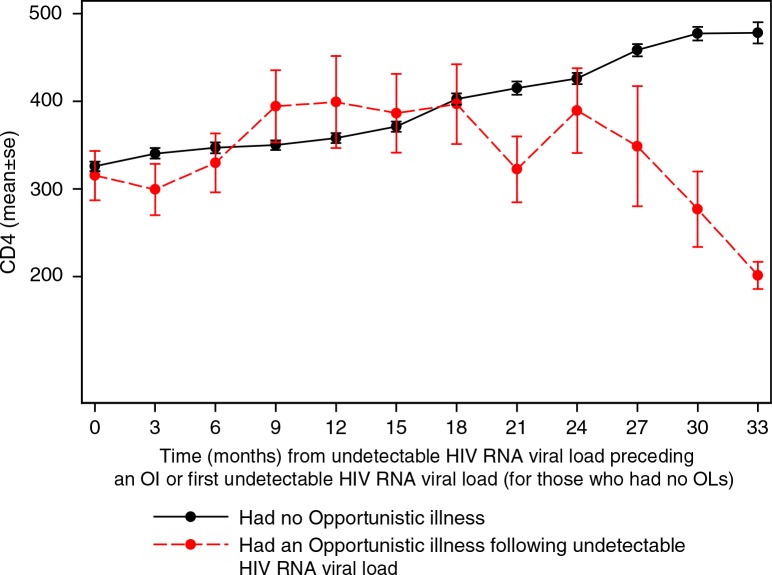
Changes in mean CD4+ T cell counts over time following an opportunistic illness/malaria or first undetectable viral load.

Figure 1 shows changes in CD4+ T cell count over time since the undetectable HIV RNA VL preceding the OI or since first detectable HIV RNA VL for those without OIs. The graph showed a decline in CD4+ T cell count in the three-month period following an episode of OI. Though the CD4+ T cell count appears to improve thereafter, the CD4+ T cell counts of those who were observed beyond one and a half years appear to continue declining among participants who had an OI.

An analysis for effects of OIs on changes in CD4+T cell count percent showed CD4+ T cell count percent to decrease at a rate of 0.04 percentage points per three months in intervals when participants had OIs, compared to an increase of 0.8 percentage points per three months in intervals when they had no episodes of OIs. However, the adjusted difference was not statistically significant (adjusted difference=1.77; 95%CI=0.93–4.48; *p=*0.198).

## Discussion

In this analysis, we found an association between having had an opportunistic illness and short-and long-term effects on HIV RNA VL and CD4+ T cell count among people taking ART. Participants who had an OI following an assessment in which their HIV RNA VL was undetectable had four times the odds of having a detectable HIV RNA VL in the following three-month assessment compared to when there was no episode of an OI (OR=3.8; 95% CI=1.7–8.4). In addition, participants had a mean decline of 24.1 CD4+ T cells/µL per three months in intervals during which they had episodes of OIs compared to a mean increase of 21.3 CD4+ T cells/µL per three months in intervals during which they had no episodes of OIs. We also observed that participants who had episodes of OIs tended to have declines in CD4+ T cell count in the long run (figure 1).

Though our results on elevation of VL were based on a small number of OIs and elevated VLs, our findings are similar to what has been observed among HIV-positive people not taking antiretroviral therapy [3–7]. While HIV RNA VL is a predictor for increased risk of OIs, a number of studies have previously demonstrated increases in HIV RNA VL following OIs among HIV-positive individuals who were not on antiretroviral therapy [13,14]. It has also been shown that infection of tissue macrophages by OIs increases their production of HIV virus [15].

The observed effects of OIs on VL underscore the importance of OI prevention interventions even among people taking ART. Prior to the availability of ART, data on the benefits of cotrimoxazole prophylaxis regardless of CD4+T cell count influenced Uganda Ministry of Health and WHO policy [12,16,17]. Similar benefits may be seen in people with HIV taking ART. For example, insecticide-treated bed nets reduced the incidence of malaria even among patients taking ART and cotrimoxazole prophylaxis [18], and stopping cotrimoxazole prophylaxis in people taking ART with high CD4+ T cell counts was associated with increased rates of malaria and diarrhoea in a randomized trial [19].

In addition to being a predictor of disease progression, HIV-1 RNA concentration in the blood is also a strong determinant of both sexual and mother-to-child transmission of HIV [13,14]. Therefore, besides independently increasing the risk of death [8,9], occurrence of OIs may also increase the risk of HIV transmission through their effects on HIV RNA VL. Thus, efforts to prevent OIs are important in both the care of those who have HIV and prevention of HIV transmission.

It is possible that we may have underestimated the association of OIs with subsequent VL elevation and CD4+ T cell count decline because the study population were visited weekly at their home and most new infections were likely to be promptly treated. It is possible that some VLs became detectable after OIs but resolved prior to the next quarterly blood draw due to prompt treatment of OIs. Many patients taking ART in programmatic settings in sub-Saharan Africa do not have access to prompt medical attention, and the effect could be greater than what we have observed in the context of prompt treatment of OIs.

In conclusion, our findings show that episodes of OIs among HIV-infected adults with suppressed VL while taking ART were associated with elevations in VL and reduced improvement in CD4+ T cell counts. Prevention of OIs is therefore important even among patients on ART who have attained suppressed VLs.
